# Inflammasome-Dependent Coagulation Activation in Sepsis

**DOI:** 10.3389/fimmu.2021.641750

**Published:** 2021-03-16

**Authors:** Runliu Wu, Nian Wang, Paul B. Comish, Daolin Tang, Rui Kang

**Affiliations:** Department of Surgery, University of Texas Southwestern Medical Center, Dallas, TX, United States

**Keywords:** inflammation, inflammasome, sepsis, DIC, coagulation

## Abstract

Sepsis is a potentially life-threatening, pathological condition caused by a dysregulated host response to infection. Pathologically, systemic inflammation can initiate coagulation activation, leading to organ dysfunction, and ultimately to multiple organ failure and septic death. The inflammasomes are cytosolic multiprotein signaling complexes that control the host response to diverse pathogen-associated molecular patterns (PAMPs) from microorganisms as well as damage-associated molecular patterns (DAMPs) from dead or dying host cells. Recent studies highlight that the activation of canonical and non-canonical inflammasomes not only mediate the maturation and secretion of interleukin-1 (IL1) family cytokines, but also trigger the release of coagulation factor III, tissue factor (F3, best known as TF) in activated macrophages and monocytes. These emerging functions of inflammasomes in immunocoagulation are further positively regulated by stimulator of interferon response cGAMP interactor 1 (STING1, also known as STING or TMEM173, a hub of the innate immune signaling network) and high mobility group box 1 (HMGB1, a nuclear DAMP). This mini-review will discuss the regulation and function of inflammasome-dependent coagulation activation in sepsis.

## Introduction

Sepsis is a challenging clinical syndrome characterized by life-threatening organ dysfunction or failure due to the dysregulated host immune response to pathogen infection, including bacteria, viruses, and fungi (1). The typical pathological process of sepsis involves the early hyperinflammatory state and the late immunosuppressive stage. This dynamic change of the host immune response is closely related to local or systemic coagulation abnormalities (2). Disseminated intravascular coagulation (DIC) is a common complication of sepsis, characterized by systemic activation of the coagulation cascade with microthrombosis, platelet consumption, and subsequent clotting factor exhaustion (3). Clinical studies have shown that the mortality rate of septic shock patients with DIC is twice that of septic patients without DIC (4), highlighting the importance of understanding the pathogenesis, diagnosis, and treatment of DIC in sepsis.

Cells of the innate immune system, such as macrophages, monocytes, neutrophils, and dendritic cells, are the first line of defense against foreign pathogens. However, excessive activation of these professional phagocytes may lead to inflammation, immune dysfunction, and abnormal blood clotting. Inflammasomes are multiprotein intracellular complexes that detect the components of microorganisms [namely pathogen-associated molecular patterns (PAMPs)] and endogenous danger signals released by injured cells [namely damage-associated molecular patterns (DAMPs)] using various pattern recognition receptors (PRRs) (5). Generally, according to whether caspase-1 (CASP1) or caspase-11 (CASP11 in mouse, also known as CASP4 and CASP5 in humans) is activated, inflammasomes can be divided into canonical and non-canonical subtypes (6, 7). Although they play an important role in host immune defense, the vigorous activation of inflammasomes also cause detrimental consequences, providing the pathogenicity of disease, including septic shock (8). In contrast, genetic depletion of core components of inflammasomes, such as *Nlrp3, Casp1, Casp11*, and gasdermin D (*Gsdmd*), protects against septic shock (9–15) or lethal endotoxemia (7) in mice, turning them into a promising target for treatment of sepsis.

In this mini-review, we introduce the types and activation of inflammasomes, discuss their roles in coagulation and thrombosis, and highlight their implications in sepsis.

## Types and Activation of Inflammasomes in Sepsis

Inflammasomes typically contain a sensor (cytosolic PRRs), an adaptor [apoptosis-associated speck-like protein containing a caspase recruitment domain (ASC)], and a zymogen (pro-CASP1) (16). Assembly of inflammasome is initiated when PRRs sensing PAMPs, DAMPs, or stress signals. Certain PRRs then recruit ASC, a bipartite protein that bridges the sensors and the effector pro-CASP1 (17). Pro-CASP1 is subsequently cleaved into active caspase, ultimately leading to maturation and secretion of interleukin 1 (IL1) family cytokines (such as IL1B and IL18) or cleaved GSDMD-mediated pyroptosis. Functionally, pyroptosis is a form of pro-inflammatory cell death. Although pyroptosis has been found to occur in various immune and non-immune cells, it was first discovered in macrophages during bacterial infections (18). GSDMD-formed pores not only mediate pyroptosis, but also facilitate the release of IL1B in a pyroptosis-independent manner (19, 20). Below, we summarize the main types of inflammasomes related to sepsis.

### Canonical Inflammasome

#### The NLRP3 Inflammasome

The most extensively studied inflammasome is the NLR family pyrin domain containing 3 (NLRP3) inflammasome, which is activated by a variety of stimuli, including PAMPs, DAMPs, pore-forming toxins, crystals, and nucleic acid (21). Of note, the basic expression of NLRP3 and pro-IL1B in macrophages is very low, and a priming signal (such as TLR ligands or IFN) is required to activate the NF-κB pathway to upregulate the expression of the components for NLRP3 inflammasome in macrophages (22). The second signal triggers NLRP3 activation by multiple mechanisms, including potassium (K^+^) efflux, increased calcium (Ca^2+^) signaling, mitochondrial translocation of NLRP3, excessive mitochondrial reactive oxygen species (ROS) generation, release of mitochondrial DNA and cardiolipin, and lysosomal leakage of cathepsins into cytosol (5, 23). Many studies have found that inhibiting the activation of NLRP3 inflammasome has a protective effect on septic animals (24). In particular, the NLRP3 inhibitor MCC950 attenuates multi-organ injuries in septic rats (25), highlighting the potential of using NLRP3 inhibitors in the treatment of sepsis.

#### The NLRC4 Inflammasome

The NLRC4 inflammasome responds to more stringent types of stimulation. NLRC4 forms a complex with certain NLR family apoptosis inhibitory protein (NAIP) family proteins, which directly bind to the NLRC4-activating ligands. For example, mouse Naip1 or Naip2 binds to the needle protein or rod component of bacterial type III secretory system (T3SS), respectively (26, 27). Moreover, both mouse Naip5 and Naip6 can recognize bacterial flagellin (27, 28). In humans, only one NAIP homolog has been identified to recognize the needle structure of T3SS. Once bound to their ligands, NAIPs oligomerize with NLRC4 to form the NLRC4 inflammasomes, leading to CASP1 activation. *In vivo*, a severe systemic inflammation is caused by activating NLRC4 inflammasomes with flagellin in monocytes, macrophage and neutrophils (29). Systemic coagulation and massive thrombosis are induced by T3SS infection in mice through the activation of inflammasome, possibly the NLRC4 inflammasome (30). Therefore, inappropriate NLRC4 activation may result in detrimental consequence in sepsis.

### Non-canonical Inflammasome

Clinically, septic shock is a multi-step process and mainly related to Gram-negative bacterial infection. Lipopolysaccharides (LPS), the main component of the outer membrane of Gram-negative bacteria, is a prototypical PAMP for studying innate immune response. Historically, the activity of LPS was determined by the membrane receptor toll-like receptor 4 (TLR4). Recent breakthroughs confirmed that CASP11 can act as a receptor for cytoplasmic LPS, which is independent of TLR4 (31, 32). The activation of CASP11 inflammasome also can promote CASP1-dependent IL1B and IL18 production by triggering the activation of NLRP3 inflammasome. CASP11 induces CASP1-independent pyroptosis, which still requires the production of cleaved GSDMD at the N-terminus (termed GSDMD-N) and subsequent translocation of GSDMD-N to the cell membrane (7). Similar function of human non-canonical inflammasome has been identified by the deletion of *CASP4* or *CASP5* in human macrophage, which impairs pyroptosis and NLRP3 inflammasome-mediated cytokine release (33–36). The contribution of non-canonical inflammasome to sepsis has been reported in septic mice model (7, 37–39). The deletion of *CASP11* or using CASP11-targeting inhibitor (e.g., oxPAPC) protects mice against LPS-induced lethality (7, 38). In addition, transgenic expression of *CASP4* in *Casp1*^−/−^*/11*^−/−^ mice renders increased susceptibility to LPS-induced shock (40), indicating the pathogenetic role of human non-canonical inflammasome in sepsis.

## Modulation and Function of Inflammasome in Coagulation

**Figure 1:**
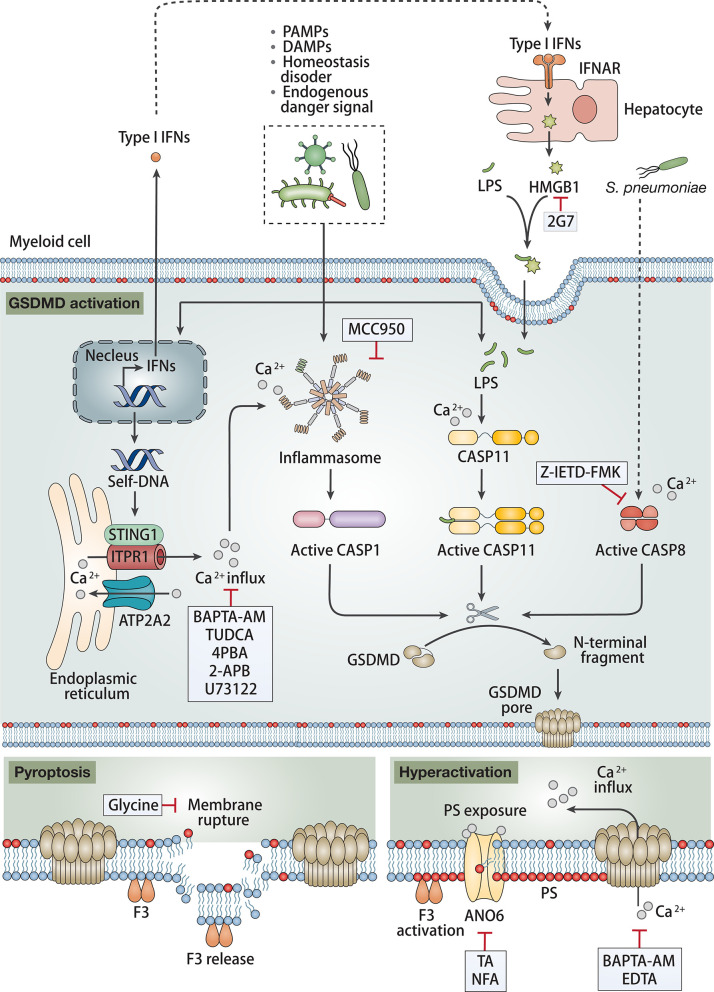
Role of inflammasome in sepsis-induced coagulation. Canonical and non-canonical inflammasome complexes in myeloid cells are assembled when pattern recognition receptors (PRRs) sense pathogen-associated molecular patterns (PAMPs), damage-associated molecular patterns (DAMPs), altered cellular homeostasis or endogenous danger signals caused by infection during sepsis. Functional inflammasome activates caspase-1 (CASP1), caspase-11 (CASP11) or caspase-8 (CASP8) to cleave gasdermin D (GSDMD) to produce N-terminal fragments (GSDMD-N). GSDMD-N forms pores on the plasma membrane, resulting in cell membrane rupture and pyroptosis or rendering cells into hyperactivation state. Coagulation factor III (F3) released from ruptured membrane promotes blood clotting. Elevated Ca^2+^ influx from extracellular space through GSDMD-N-formed pores in hyperactivation state promotes phosphatidylserine (PS) exposure, thereby enhancing the pro-coagulant activity of F3. Type I interferons (IFNs) mediates release of hepatocyte high mobility group box 1 (HMGB1), which facilitates LPS entering cytosol. Stimulator of interferon response cGAMP interactor 1 (STING1) senses infection-induced DNA damage and mediates CASP1/11/8 activation. Inhibition of inflammasome activation and subsequent pyroptosis prevents sepsis-induced coagulation.

Most patients with sepsis show hemostatic changes, while DIC occurs in ~35% of patients, resulting in organ dysfunction and death (41). The most principal initiator of coagulopathy in sepsis is coagulation factor III (F3). It is a transmembrane single-chain glycoprotein composed of 263 amino acid residues, with a molecular weight of about 47 kDa (42). F3 initiates the blood coagulation cascade by binding to coagulation factor VII/VIIa (F3:VIIa complex) on the cell surface (42). During sepsis, the host immune response to PAMPs (such as LPS) rapidly triggers the activation of coagulation by inducing the expression of F3 on monocytes, platelets or endothelial cells (43–46). Additionally, the mechanism of regulating F3 activity by transforming F3 from an inactive state to an active state (a process called F3 decryption) also contributes to coagulation activation. Exposure of anionic phospholipids, such as phosphatidylserine (PS), on the outer leaflet of the plasma membrane is considered to be the main cause of F3 decryption (47). This process optimizes the presentation of F3:VIIa complex to provide more efficient binding sites to their substrates factors IX and X. Increased PS exposure on the surface of circulating leukocytes is observed in sepsis (47). Genetic deletion of *F3* or blocking F3 activity using neutralizing antibodies in sepsis animal models prevents activation of coagulation and decreases the mortality (30, 48, 49). The administration of PS-neutralizing binding protein lactadherin markedly ameliorates sepsis-induced coagulation and lethality (50). These evidences suggest that treatment of the altered coagulation would be a reasonable approach to improve the mortality of sepsis. Some DAMPs, such as cell-free DNA, histones, heat shock proteins, and high mobility group box 1 protein (HMGB1) (51, 52), have been reported to induce coagulopathy in sepsis by furtherly augmenting systemic inflammation (53) or impairing the activation of anticoagulants (e.g., protein C) (54). These DAMPs may be released from damaged cells due to apoptosis, necroptosis, or pyroptosis, but the contribution of cell death to coagulation in sepsis is context-dependent. Recently, three independent groups found that both CASP1 and CASP11-dependent inflammasomes trigger systemic coagulation in mice through GSDMD-N-mediated increased F3 release or F3 activity in macrophages and monocytes (Figure 1).

### Caspase Activation

Either CASP1 or CASP11 can promote the coagulation cascade, depending on the type of bacterial infection. CASP1 is required to release active F3 in mouse bone marrow-derived macrophages (BMDM) challenged or stimulated by bacterial T3SS inner rod protein, including EprJ and EscI from Escherichia coli (E.coli), BsaK of Burkholderia pseudomallei, and PrgJ of Salmonella typhimurium (30). Similarly, T3SS treatment or *E. coli* infection induces CASP1-dependent F3 release in THP1 cells, a human monocytic cell line derived from an acute monocytic leukemia patient (30). *In vivo*, lack of *Casp1* (instead of CASP11) protects mice from EprJ-induced lethality associated with the reduction of DIC biomarkers in blood (30). In polymicrobial sepsis induced by cecal ligation and puncture (CLP), inhibiting the activity of CASP1 with the NLRP3 inhibitor MCC950 also reduces platelet activation in rats (25, 55). These animal studies suggest that CASP1 has a potential role in regulating septic coagulation in mice and rats.

The activation of CASP11-dependent inflammasomes also mediates the release or activation of F3 in sepsis caused by CLP, *E. coli* infection, bacterial outer membrane vesicle (OMV) infection, and LPS stimulation *in vivo* (56, 57). Similar to clinical anticoagulant heparin treatment, in the lethal endotoxemia mouse model, the absence of *Casp11* inhibits the activation of coagulation (56, 57). In *Casp11*-deficient mice, systemic coagulation triggered by the initiation of poly(I:C) and subsequent LPS administration is also blocked (30, 56). Extracellular HMGB1 is not only a DAMP, but also a carrier that brings LPS into the intracellular space (58). In particular, extracellular HMGB1 from liver mediates LPS uptake and promotes the externalization of phosphatidylserine (PS), which is important for F3 activation in macrophages. In contrast, depletion of *Casp11* limits HMGB1/LPS-induced PS exposure and subsequent F3 activation in macrophages (59). Therefore, CASP11-dependent inflammasome is an important regulator of F3 release and activation in macrophages. While TLR4 is essential for LPS-induced gene expression of *Casp11*, TLR4 is considered to be dispensable in most inflammasome-mediated coagulation (30). Injection of LPS primed with poly(I:C) also induces coagulation cascade in *Tlr4*-deficient mice (56). The function of human CASP4 or CASP5 in sepsis-induced coagulopathy remains poorly understood, but it will be enlightened by these investigations of CASP11 in mouse models.

In certain bacterial infections (especially *Y. pestis* and *Y. pseudotuberculosis*), the apoptotic non-inflammatory caspase CASP8 also participates in inducing pyroptosis by activating the NLRP3 inflammasome (60) or acting as a structural component of the inflammasome (61). Consequently, CASP8 (but not CASP1 or CASP11)-mediated GSDMD-N production is required for F3 release in BMDM during *Streptococcus pneumoniae* (*S. pneumoniae*) infection (62). Collectively, these studies indicate that inhibition of caspase activation may have a potential therapeutic effect on fatal coagulopathy during sepsis.

### GSDMD Cleavage

The activation of CASP1, CASP11, or CASP8 causes the cleavage of GSDMD, thereby generating a pyroptotic p30 fragment, namely GSDMD-N. GSDMD-N-mediated pore formation has been regarded as the terminal event of pyroptosis or hyperactivation state. Genetic or pharmacological inhibition of GSDMD expression or cleavage prevents F3 release or activation *in vitro* or systemic activation of coagulation in mice induced by CLP, *E.coli* infection, bacterial rod proteins or OMVs stimulation, as well as LPS challenge in the absence or presence of HMGB1 (30, 56, 57, 59, 62). GSDMD-mediated F3 release is pyroptosis-dependent. Glycine (an osmotic protectant) inhibits the release of F3 in EprJ-infected BMDM by pyroptosis-driven cell membrane rupture instead of GSDMD-mediated pore formation (30). Although the purinergic receptor P2X7 (P2RX7) has been shown to mediate pyroptosis in a GSDMD-independent manner (63), it seems that P2RX7 is not required for the coagulation cascade in endotoxemic mice (30, 56).

It is worth noting that the GSDMD-mediated coagulation cascade may occur in a pyroptosis-independent manner. Glycine is unable to affect F3 activation in mouse peritoneal macrophages (PM) stimulated by cytoplasmic LPS, suggesting another mechanism independent of pyroptosis. Alternatively, the pores formed by GSDMD render cells into a hyperactivation state, which is adequate to permit Ca^2+^ influx, thereby promoting PS exposure through Ca^2+^-dependent scramblase anoctamin 6 (ANO6). After the externalization of PS is increased, the activity of F3 is enhanced after LPS challenge *in vivo* and *in vitro*, which can be attenuated by using specific PS binding proteins, such as lactadherin and MFG-E8 (56). These studies describe a direct link between GSDMD and coagulopathy, although its mechanism of action is stimulus-dependent.

Cell membrane rupture also occurs in necroptosis, a form of regulated necrosis depending on several kinases, including receptor interacting protein kinase 1 (RIPK1). RIPK1 expressed in epithelial cells favors tumor necrosis factor (TNF)- or TNF/Z-VAD-FMK-induced coagulation with increased plasma F3 in mice (64). These findings indicate that multiple types of necrosis contribute to coagulation through different mechanisms.

### STING1 Activation

Stimulator of interferon response cGAMP interactor 1 (STING1) is an ER-associated membrane protein and plays a complex role in innate immune sensing of pathogens. Excessive activation of STING1 pathway is involved in pathogenesis of sepsis and is recently reported to drive lethal coagulation in sepsis through GSDMD-dependent mechanism. STING1, coupled with inositol 1,4,5-trisphosphate receptor type 1 [ITPR1, a calcium release channel of endoplasmic reticulum (ER)] and the ATPase sarcoplasmic/ER Ca^2+^ transporting 2 (ATP2A2, a calcium uptake pump of ER), mediates cytosolic calcium influx to activate CASP1, CASP11, or CASP8 in macrophages/monocytes in response to different infections (62). Therefore, *Sting1* depletion limits the production of GSDMD-N in THP1 cells mediated by CASP1/11/8, resulting in a decrease in F3 release. Reduced coagulation activation and prolonged animal survival are observed in septic mice (CLP, *E.coli* and *S. pneumoniae* infection) with conditional deletion of *Sting1* in myeloid cells (62). Moreover, mRNA expression of *STING1* and *GSDMD* in peripheral blood mononuclear cell (PBMC) closely correlates with DIC severity in patients with sepsis, highlighting the regulatory role of STING1 in DIC during sepsis (62). Notably, STING1-mediated type I interferon (IFN) response does not seem to be important for inflammasome-mediated coagulation response during sepsis, because deletion of type I IFN receptor (*Ifnar*) or interferon regulatory factor 3 (*Irf3*) in mice fails to block infection-induced coagulation activation (62). However, another study suggests that IFNs may contribute to coagulation activation due to its ability to induce hepatocyte HMGB1 release, leading CASP11-dependent GSDMD activation and PS exposure (59). Further animal experiments are needed to understand the role of IFN-dependent HMGB1 release in blood coagulation.

### Ca^2+^ Influx

Increased cytosolic Ca^2+^ influx, either released from ER or entered extracellularly through calcium channels, is a critical signal for immune response (65, 66), including modulating inflammasome activation (67–70). Inhibiting cytosolic calcium accumulation by calcium chelator (BAPTA-AM and EDTA) or ER stress inhibitor (TUCDA and 4PBA) leads to reduced F3 release or activity in THP1 or murine macrophages (56, 62). Similarly, decreased Ca^2+^ released by TUCDA or Ca^2+^ channel modulator (2-APB) protects against coagulation activation in CLP mice. (56). In contrast, raising Ca^2+^ influx by ER stress agonist (tunicamycin and thapsigargin) (62) or calcium inophore (A23187) (56) promotes F3 release or activity. These drug studies support the function of cytosolic Ca^2+^ influx in mediating coagulation activation during sepsis. In addition, the production of GSDMD-N in THP1 or BMDM during inflammasome activation is also inhibited by blocking cytosolic Ca^2+^ influx using the knockdown of *ITPR1*, overexpression of *ATP2A2* or inhibition of phospholipase C gamma 1 (PLCG1) (62). Moreover, extracellular Ca^2+^ also enters through GSDMD-N-formed pores to trigger coagulation cascade by promoting PS exposure (56). In general, these findings suggest that during sepsis, Ca^2+^ influx can act as both a regulator and an effector of inflammasome activation during septic coagulation. Approaches that control the Ca^2+^ concentration may improve the therapeutic effect of anticoagulation.

## Conclusion and Outlook

The molecular mechanisms of how systemic coagulation is triggered by the inflammasome during lethal sepsis brings a new understanding of the inflammasome function and sets a new stage for immunocoagulation studies. However, some questions have raised and remain unsolved. First, it is not yet clear how different types of inflammasomes coordinate to regulate the coagulation response, because clinical sepsis is usually caused by polymicrobial infection. Second, most studies have focused on the direct effects inflammasomes have on the release and activation of F3. However, whether F3 in turn regulates inflammasome activation is still unknown. Third, how to transform these new understandings into treatment of inflammasome-dependent coagulation during sepsis in human patients? Since the treatment with anticoagulant after onset of sepsis has not resulted in improved clinical outcomes, administration or combination of inflammasome-associated inhibitors may be a favorable approach to fight against sepsis-induced coagulation. Some drugs have displayed a promising effect to protect inflammasome-related coagulation during sepsis (Table 1). The existing small molecules that block inflammasome activation could also be investigated for their potential role in controlling coagulation.

**Table 1:** Potential inhibitors of inflammasome-dependent coagulation.

**Mechanism**	**Function**	**Name**	**Usage**	**Model**	**References**
Reduce Ca^2+^ influx	Calcium chelator	BAPTA-AM	Up to 10 μM	PMs WT or *ITPR1*-KD BMDMs/THP1	(56, 62)
		Ethylenediaminetetraacetic acid (EDTA)	Up to 600 μM	PMs	(56)
	ER stress inhibitor	Tauroursodeoxycholic acid (TUDCA)	200 mg/kg	WT or *Tmem173^−/−^* mice	(62)
			50 μM	THP1	(62)
		4-phenyl butyric acid (4PBA)	1 mM	THP1	(62)
	D-myo-inositol 1,4,5-trisphosphate (IP_3_) receptor antagonist	2-Aminoethoxydiphenylborane (2-APB)	20 mg/kg	WT or *Tmem173^−/−^* mice	(62)
	TMEM16F inhibitor	Tannic acid (TA)	NA	PMs	(56)
		Niflumic acid (NFA)	NA	PMs	(56)
	PLCG1 inhibitor	U73122	10 μM	THP1	(62)
			30 mg/kg	WT or *Gsdmd^105*N*/105*N*^* mice	(62)
Inhibit caspase 8 cleavage	Caspase 8 inhibitor	Z-IETD-FMK	20 μM	WT or *Casp1^−/−^Casp11^−/−^* BMDMs	(62)
Prevent NLRP3 oligomerization	NLRP3 inhibitor	MCC950	50 mg/kg	Rat	(25)
Delete *in vivo* macrophage	Macrophage remover	Clodronate liposomes	40 mg/kg	Mice	(30, 56)
Neutralize HMGB1	HMGB1 antibody	2G7	160 μg/mouse	Mice	(56)
Prevent membrane rupture	Osmoprotectant	Glycine	5 mM	BMDMs	(30)

Regulated inflammasome activity is still essential for host defense against pathogens because mounting the immune response with its associated secretory cytokines would further contribute to the adaptive immune response. Thus, treatment of sepsis-induced coagulation by inhibiting inflammasome activity should be strictly monitored to avoid severe side effects caused by a suppressed immune response. Therefore, an in-depth understanding of the mechanism of coagulopathy triggered by inflammasomes is essential for identifying new therapeutic targets and developing more beneficial therapies.

## Author Contributions

RK and DT conceived of the topic for this review. All authors listed have made a substantial, direct and intellectual contribution to the work, and approved it for publication.

## Conflict of Interest

The authors declare that the research was conducted in the absence of any commercial or financial relationships that could be construed as a potential conflict of interest.
